# Talking with consumers about energy reductions: recommendations from a motivational interviewing perspective

**DOI:** 10.3389/fpsyg.2015.00252

**Published:** 2015-03-13

**Authors:** Florian E. Klonek, Simone Kauffeld

**Affiliations:** Department of Industrial/Organizational and Social Psychology, Technische Universität BraunschweigBraunschweig, Germany

**Keywords:** change intervention, change talk, energy-saving, interaction analysis, motivational interviewing, MI Skill Code, resistance to change, R-index

## Abstract

Reduction of energy costs has become a concern for many organizations. First, we review energy-saving studies in organizations in which consumers showed resistance to change their behavior. Second, we relate resistance to change to the psycholinguistic construct “sustain talk” that describes verbal arguments against behavior change (e.g., “Work processes have priority here”). Third, we argue how Motivational Interviewing (MI)—an interaction-approach to facilitate behavior change—might be helpful in dealing with this behavior. We transfer MI to interactions about energy-savings in organizations and demonstrate how qualification in MI for energy managers may affect these interactions. Therefore, we present three short case scenarios (i.e., video vignettes) that demonstrate socio-interactional mechanisms underlying energy-relevant decisions and behaviors. Consumer' verbal responses are graphed as one single time-variant index of readiness versus resistance (R-index) in order to illustrate interactional dynamics. In sum, we combine theoretical and empirical perspectives from multiple disciplines and discuss an innovative socio-interaction approach that may facilitate energy-efficient behavior in organizations.

Reducing the impact of rising energy costs has increasingly become a concern for many organizations (Garabuau-Moussaoui, 2014; Leyge, 2014; Morgenstern, 2014; Tharan, 2014). Whereas the engineering field has developed mostly technical measures to increase energy efficiency, there is an increasing trend to recognize that the behavior of consumers who actually work in the organizations is equally important (Kraft and Neubeck, 2004; Carrico and Riemer, 2011; Janda, 2011; Steiner et al., 2011; Kauran, 2013; Cowan, 2014; Parkes, 2014; Schäfer, 2014). However, recent studies that aim to reduce energy behavior have also reported that consumers show resistance to changing their energy consumption at work (Griesel, 2004; Kaplowitz et al., 2012; Murtagh et al., 2013).

In order to facilitate interactions with consumers about energy-relevant decisions, this perspective paper offers the following contributions. First, we give a brief overview of organizational studies that reported consumer resistance when energy-relevant behaviors were in need of change. Second, we relate resistance to change to the psycholinguistic constructs of change language (i.e., change talk vs. sustain talk) and hypothesize that language is an active ingredient in fostering behavior change. Third, we propose Motivational Interviewing (MI) to be a social interaction-based approach that may help energy managers to promote energy-saving behaviors in organizations. We draw on empirical evidence in the field of clinical psychology (e.g., Magill et al., 2014) in order to hypothesize how interactional mechanisms could affect energy-relevant behavior. Fourth, we produced demonstration material that highlights how MI-based approaches could be applied in the context of energy-saving interventions. Fifth, we visualize interactional dynamics with a newly developed temporal measure of readiness and resistance to change by means of three video vignettes. Human resources and training departments in organizations can use these vignettes for training purposes.

## Behavior-based energy-saving interventions and resistance to change

Organizations are increasingly trying to save energy either for economic purposes or to accomplish a reduction of carbon emissions (Homburg, 2004; D'Mello et al., 2011; DuBois et al., 2013). Whereas one energy-efficiency approach includes technical improvements, such as increased heat insulation or replacing ventilation with volume flows (Lutzenhiser, 1993), technical engineers are realizing that the behavior of people who work in organizations also contributes to energy consumptions (Janda, 2011). Science laboratories in universities have one of the highest energy usages and offer high potential to implement behavior-based energy-conservation procedures with consumers (Kaplowitz et al., 2012). Kaplowitz et al. interviewed 59 participants (principal investigators, lab staff, and student researchers) about possibilities to adapt energy-conserving behavior at work. Despite a favorable attitude toward energy-saving behaviors, participants argued that operational, economic, and work-related barriers hindered them from saving energy. In a study from Griesel (2004), the author conducted a workshop with university staff to promote energy-efficient behavior. She reported that some of the proposed measures (e.g., switching off laptop computers during breaks) were considered unacceptable by participants.

Murtagh et al. (2013) reported similar problems in an energy-saving intervention study. The authors implemented a monitoring device in a university office building that displayed employees their current energy use. Unfortunately, 41% of the participants did not even register for the feedback device. The authors also held focus groups about their office energy use and reported that participants showed a “syndrome of reasoning” (Murtagh et al., 2013, p. 724)—a term for describing verbal responses or self-defense for not saving energy (e.g., inconvenience, technical reasons/myths, social norms, automation, work demands, savings are too small, etc…).

Altogether, observations from these organizational studies suggest that the language of consumers seems to be indicative of their respective motivation. Participants who are not motivated to take further actions will also express this verbally. In fact, verbal behavior (“I will not do this”) is a powerful means to express resistance toward change measures (Klonek et al., 2014). More recent methodological work has proposed measuring participants' readiness or resistance^1^ in interactions about behavior change in terms of change talk versus sustain talk (Moyers et al., 2007; Miller and Johnson, 2008; Gaume et al., 2010; Magill et al., 2010; Klonek et al., 2014; Lombardi et al., 2014; Paulsen et al., 2015). Change talk includes statements that express consumers' readiness to adopt energy-saving routines, such as reasons (“Energy waste is related to increased department costs”), desires (“We do not want to waste energy here”), or needs to change (“It is prohibited by security management to open the windows at night”). By contrast, sustain talk comprises language that speaks against energy-saving measures, such as resistance, reasons to sustain the status quo, a lack of abilities (“I do not know how to operate the heating system—therefore, I don't change it”), or lack of commitment (“I won't promise that I will always think about switching off the lights”). Furthermore, change and sustain talk can be regarded as driving and hindering forces that may determine consumers' energy-related behaviors (Klonek et al., under revision).

This language-based view takes into account that consumers usually express ambivalence rather than sole resistance toward change measures (“Yes, energy savings are important, *but* it impedes my work flow to shut down the computer during breaks”; Piderit, 2000; Arkowitz, 2002; Klonek et al., 2014). In this view, one part of the statement argues in favor of change, whereas the other part argues against change. These conflicting values are like opposite sides of a decisional balance (Janis and Mann, 1977; Klonek et al., under revision) that are dynamically tipping from one side (sustaining behavior) to the other (changing behavior). So what can organizations do in order to increase the weight of the decisional balance so that consumers move toward saving energy?

One social interaction-based approach that makes use of an individual's ambivalence toward change is a method called MI. It is a communication-based approach that has received large support by numerous meta-analyses as an evidence-based intervention in the helping professions (Hettema et al., 2005; Rubak et al., 2005; Lundahl et al., 2010; Magill et al., 2014). We will briefly present the basic tenets of MI and argue how energy managers in organizations could benefit from MI training.

## What is motivational interviewing and how might it improve interactions about energy behavior?

MI can be considered as a social interaction-based approach that combines a humanistic mindset with verbal micro-techniques. Technically, it is defined as a

“collaborative, goal-oriented style of communication with particular attention to the language of change. It is designed to strengthen personal motivation for and commitment to a specific goal by eliciting and exploring the person's own reasons for change within an atmosphere of acceptance and compassion.” (Miller and Rollnick, 2013, p. 29).

MI shares some common ground with participatory energy-saving interventions (e.g., consumer-centered formats, such as workshops; Matthies, 2000; Griesel, 2004) and commitment-building strategies (Lokhorst et al., 2013) that have been proposed to be effective in promoting sustainable behavior change. However, MI significantly contributes to these approaches because it gives clear recommendations for how to deal with resistance and how to increase intrinsic motivation. For example, one of the MI principles is to work out discrepancies in a collaborative way: These discrepancies can encompass, for example, energy-wasting behaviors that are at odds with specific values of the consumer (e.g., “economizing resources” or “being a role-model”). An MI approach advocates that energy-saving procedures should not be enforced top-down from the organization, but rather that consumers' intrinsic motivation has to be developed bottom-up. MI also adds a goal-oriented component in the interaction by reinforcing consumers' own argumentation to save energy, in essence, tipping the decisional balance toward a specific target behavior (i.e., saving energy).

Traditionally, MI is taught within the helping professions, for example, among therapists, counselors, physicians, or nurses (Madson et al., 2009; Forsberg et al., 2010; de Roten et al., 2013). More recent studies have provided evidence that MI is also teachable to non-helping professions, e.g., for engineers (Klonek and Kauffeld, in press) or for environmental inspectors (Forsberg et al., 2014). Whereas MI has not been used in organizations in order to reduce energy-related behavior at work, it has great promise of equipping energy managers successfully with the right mindset and verbal skills in order to discuss these matters.

## Demonstration of MI: the energy manager as a social change agent

In order to showcase the use of MI as a communication method for energy managers, we developed three vignettes (i.e., scripted audio and video material) in which an energy manager discusses energy-efficient behavior with an employee. The development of this material was guided by a multi-step procedure in which we integrated interaction material from two different sources.

First, we used three existing interaction scenarios that systematically varied in terms of MI consistency (Project MILES, 2011)^2^. These scenarios were developed independently from a German MI expert who is also a member of the Motivational Interviewing Network of Trainers (2008). Transcripts were also annotated previously with a coding instrument that classifies verbal behaviors in MI (Martin et al., 2005; Hannöver et al., 2013). As the content of these interactions was not related to energy-saving behavior (i.e., the conversations covered the reduction of smoking behavior), we only used the structure of the behavioral dynamics and replaced the content with arguments that are characteristic of energy-related interactions.

The second source of data included videotaped interactions in which energy advisors discussed energy-reduction measures with consumers in non-residential buildings (cf. Klonek et al., 2014, submitted). These videotapes served to provide typical arguments that are provided within energy-related interactions (e.g., replacement of several refrigerators with a single one).

Material from both sources was combined systematically in an iterative process and resulted in three vignettes about energy reductions at work (see supplementary audio online material, “Audio 1–3” for English conversations; “Audio 4–6” for German conversations). Table 1 shows the first seven turn takes for each scenario. We kept the content of each conversation similar but varied the interactional dynamics in each conversation in order to illustrate how subtle micro-behaviors may influence the course of an interaction (i.e., the subsequent response of the conversational partner). The mechanisms of interpersonal dynamics were based on theoretical assumptions (i.e., technical hypotheses) and empirical support from MI research (Magill et al., 2014): The main assumption of the technical hypothesis is that MI inconsistent behaviors are positively associated with sustain talk (e.g., Apodaca and Longabaugh, 2009; Klonek et al., 2014) and negatively associated with change talk (e.g., Gaume et al., 2010), whereas MI consistent behaviors make change talk more likely and sustain talk less likely (e.g., Moyers et al., 2007). With respect to conversations about energy-reductions at work, the technical hypothesis implies that energy managers who are trained in MI verbal skills (Energy Manager B; Audio 2 and 4) will likely increase employees change talk. In contrast, energy managers that have not acquired verbal skills in MI (Energy Manager C; Audio 3 and 6) will also use more MI inconsistent behaviors in conversations to reduce energy and therewith decrease change talk and/or increase sustain talk, respectively.

**Table 1:** **Comparison of the first seven turn-takes from the three demonstration interactions about energy-saving behavior at work (fully coded transcripts of all scenarios are provided as online material)**.

**Event**	**Speaker**	**Scenario A–C**		
		**Energy manager A**	**Energy manager B**	**Energy manager C**
1	Energy manager	Today, I would like to talk with you about possibilities to save energy. [Structure]		
2	Employee	Okay. [Follow Neutral]		
3	Energy manager	You work in a laboratory. There are some options that will certainly allow you to save energy. [Giving Information]		
4	Employee	[I not only work in a laboratory, but I also work in an office.] [Follow Neutral] [There are certainly some options to save energy.] [Change Talk-Other] [These so-called "options" are always connected to large expenditures.] [Sustain Talk-Reason]	[I not only work in a laboratory, but I also work in an office.] [Follow Neutral] [There are certainly some options to save energy.] [Change Talk-Other] [However, these so-called “options” are always connected to large expenditures.] [Sustain Talk-Reason]	[I not only work in a laboratory, but I also work in an office.] [Follow Neutral] [These so-called “options” are always connected to large expenditures.] [Sustain Talk-Reason]
5	Energy manager	**Don't be so rash**. [Confrontation] First off, we should speak about the methods you already use to save energy. [Structure] **Can you think of some?” [Closed Question]**	First we should perhaps talk about where you already save energy. [Structure] **What do you do already to save enery? [Open Question]**	**Don't be so rash**. [Confrontation] First off, we should speak about the methods you already use to save energy. [Structure] **Can you think of some? [Closed Question]**
6	Employee	[Well, for example, I have set up my PC with a coupler strip so that it is not running on standby the entire time.] [Change Talk-Taking Steps] [But if I am in a hurry in the evenings, I don't always remember to do this.] [Sustain Talk-Taking Steps]	[Well, I always turn on my PC using a coupler strip so that it is not always running on standby.] [Change Talk-Taking Steps] [But in the evenings if I am rushed before quitting time, I don't always do this.] [Sustain Talk-Taking Steps]	[Well, for example, I have set up my PC with a coupler strip so that it is not running on standby the entire time.] [Change Talk-Taking Steps] [But if I am in a hurry in the evenings, I don't always remember to do this.] [Sustain Talk-Taking Steps]
7	Energy manager	So it's not so important to you to save energy in this way. I mean, it is a hand movement - then the switch is turned off. [Confrontation]	**So often in the past you have switched the PC completely off, so that it does not run on Standby. Occasionally, though, you are in too much of a hurry and this is not consistently done**. [Complex Reflection]	So it's not so important to you to save energy in this way. I mean, it is merely a hand movement - then the switch is turned off. **That is really no big deal!** [Confrontation]

*Bold = highlights important differences in verbal behavior of the energy manager*.

## A closer look at verbal sequences in energy-related consumer interactions with the MI skill code

In a second step, we used the MI Skill Code (MISC; Miller et al., 2008) in order to shed light on the interactional dynamics of these conversations. The MISC is an observational coding instrument to assess MI specific verbal skills (for the German version, MISC-d^3^ : Klonek and Kauffeld, 2012). It defines three behavioral macro-categories for interviewer behaviors (i.e., energy manager) that are either consistent, inconsistent, or neutral to an MI approach. Along the same line, the MISC also defines three macro-categories for the interaction partner (i.e., consumer) that describe their verbal response in terms of change talk, sustain talk, or follow neutral (i.e., no relation to the change topic). Finally, all behavioral macro-categories can be classified into more fine-grained behaviors: For example, open questions, affirmations, emphasizing control, or giving support are all consistent with MI, whereas warnings and confrontations are inconsistent with MI (a full overview of all 34 codes of the MISC is given in the manuals). Altogether, the MISC can be used for annotating conversational dynamics for process researchers (e.g., Moyers et al., 2007). We coded the verbal behaviors between the energy manager and the employee (all coded transcripts, i.e., the Energy Manager A–C, are provided as supplementary online material; “Data Sheet 1–3” for English transcripts; “Data Sheet 4–6” for German transcripts). Table 1 shows the MISC codings for the first seven speaker turns in each scenario. It aims to illustrate how subtle micro-changes within a conversation could influence the motivational response of the conversational partner.

The energy managers start to differ in their verbal behavior in the fifth event of each scenario (cf. Table 1). For example, energy manager B uses an open question instead of a closed question. The assumption in MI is that open questions are person-centered verbal techniques that invite the interaction partner to disclose more information. In this case, the manager asked the employee about her past behaviors to save energy. The question is evocative because it stimulates the employee to discover potential discrepancies between behaviors at home vs. at work.

In the seventh event, the manager in scenario B uses a complex reflection—MI consistent behavior—whereas the managers in scenarios A and C confront the employee. Confrontations are MI inconsistent “expert-like responses that have a particular negative-parent quality” (Miller et al., 2008, p. 11). They restrict the autonomy of the employee and can even arouse reactance (Klonek et al., 2014). By contrast, complex reflections are person-centered techniques that repeat or paraphrase statements of the employee, but can also add meaning to it. Reflections are active listening statements in which the energy manager tries to understand the problems of the employee in implementing energy-saving routines. This can positively influence the relationship between conversational partners. Furthermore, reflections help the conversational partner to listen to their own statements (i.e., reflecting as a form of verbal mirroring) and selectively stress their change talk to direct the interaction toward the change target (Barnett et al., 2014).

## Capturing change-related dynamics with consumers: the R-index

**Figure 1:**
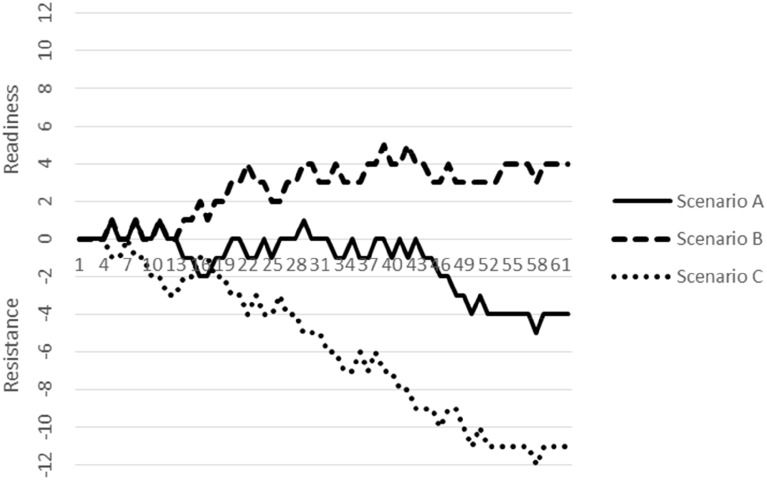
**Interactional dynamics graphed with the R-index (Readiness/Resistance) for the three demonstration scenarios on energy-saving behavior at work**. *Note*. The x-axis shows the number of events (i.e., parsed thought units or utterances).

In the previous section, we have described some micro-interactional dynamics using the MISC (e.g., MI inconsistent behavior, followed by sustain talk). Whereas this perspective helps energy managers to reflect on their verbal behaviors, we also want to show how to capture the readiness of consumers on a broader interaction scale. As noted above, the MISC defines verbal responses of conversational partners in terms of change talk, sustain talk, and follow neutral. Recent MI process research suggests using composite scores of change and sustain talk as a “single measure of motivational balance” (Magill et al., 2014, p. 7). Therefore, we developed a mathematical function that transforms these verbal codes into a single-index of readiness and resistance: The R-index (a full description is given in Klonek et al., under revision). Basically, change talk is transformed into a positive integer (+1), whereas sustain talk is transformed into a negative integer (−1), and follow neutral is transformed into zero (0). As the verbal behavior of the conversational partner unfolds over time, it creates a repeated measurement of change talk (+1) and sustain talk (−1) utterances. One of the basic idea in MI is that conversational partners can talk themselves into the target behavior (e.g., saving energy) by increasing their own change talk. Therefore, the sequence of verbal responses is cumulated from the beginning until the end of the interaction. This summation results in a time-variant index that can show readiness to change (positive slope) versus resistance to change (negative slope). We have produced R-curves for all three vignettes (A–C) as an interactive video demonstration (see video material in the supplementary online material, “Video 1–3” for English videos; “Video 4–6” for German videos). Figure 1 depicts how the readiness of the employee increases stepwise in scenario B. As noted above, energy manager B used verbal techniques that are characteristic for MI. By contrast, the R-index in scenario C indicates strong employee resistance. Equally, energy manager C showed behaviors that are inconsistent with an MI approach, such as confronting, blaming, and restricting autonomy of the employee. In scenario A, the R-index fluctuates between positive and negative values, that is, the employee showed ambivalence toward change. In this scenario, energy manager A showed both MI consistent and inconsistent behaviors.

All vignettes (coded transcripts, audio files, and videos showing the R-index) can be used for sensitizing practitioners for interactional dynamics in energy-related conversations or as MI training material. Furthermore, future studies can use this training material to investigate whether MI can help organizations to reduce energy consumption in organizations.

## Conclusion

The current perspective integrated the expertise of different disciplines, that is, clinical psychology, change management, communications, and behavioral sciences. We presented a MI-based socio-interactional approach that may positively influence energy-relevant decisions in organizations by means of its person-centered and directive approach. By creating role-played vignettes based on recent empirical meta-analyses about MI in clinical process studies (Magill et al., 2014), we illustrated how MI-consistent and MI-inconsistent employer behaviors could theoretically affect consumer responses in the context of energy-related behavior change discussions. We introduced an observational coding scheme (the MI Skill Code) as a means to investigate behavior change interactions. Finally, we created video vignettes of the coded material in which we summarized the complex coding system into one single index of consumer readiness within an energy-related conversation. This material can be used for sensitizing energy managers and change agents for interpersonal dynamics in behavior change and for future energy-saving studies that aim to use MI.

### Conflict of interest statement

The authors declare that the research was conducted in the absence of any commercial or financial relationships that could be construed as a potential conflict of interest.

## Footnotes

^1^Please note that the terms resistance and sustain talk are not interchangeable terms. Typically, current MI theory distinguishes between resistance and sustain talk. Resistance is “interpersonal behavior that reflects dissonance in the working relationship [whereas] *sustain talk* does not in itself constitute discord” (Miller and Rollnick, 2013, p. 408). Resistance is necessarily interpersonal (i.e., not residing within the person) and specifically different from sustain talk. As organizational researchers are more familiar with the term “resistance to change,” we use this term in order to connect it with recent conceptions of socio-interactional dynamics in behavior change.

^2^http://www.medizin.uni-greifswald.de/medpsych/index.php?id=453$

^3^The MISC-d also includes information on software-support, observer training material, data checking-routines, and use of neutral terms for interaction partners to make the instrument transferable to energy-saving conversations.

## References

[B51] Apodaca T. R. Longabaugh R. (2009). Mechanisms of change in motivational interviewing: a review and preliminary evaluation of the evidence. Addiction 104, 705–715. 10.1111/j.1360-0443.2009.02527.x 19413785 PMC2756738

[B1] Arkowitz H. (2002). Toward an integrative perspective on resistance to change. J. Clin. Psychol. 58, 219–227. 10.1002/jclp.1145 11793334

[B2] Barnett E. Spruijt-Metz D. Moyers T. B. Smith C. Rohrbach L. A. Sun P.. (2014). Bidirectional relationships between client and counselor speech: the importance of reframing. Psychol. Addict. Behav. 28, 1212–1219. 10.1037/a0036227 24955660 PMC4274216

[B3] Carrico A. R. Riemer M. (2011). Motivating energy conservation in the workplace: an evaluation of the use of group-level feedback and peer education. J. Environ. Psychol. 31, 1–13 10.1016/j.jenvp.2010.11.004

[B4] Cowan K. (2014). Energy steward turn off the lights or 1000 little steps, in European Conference on Behaviour and Energy Efficiency (Oxford, UK).

[B5] D'Mello S. Ones D. S. Klein R. M. Wiernik B. M. Dilchert S. (2011). “Green company rankings and reporting of pre-environmental efforts in organizations, in Poster session presented at the Annual Conference of the Society for Industrial and Organizational Psychology (Chicago, IL).

[B6] de Roten Y. Zimmermann G. Ortega D. Despland J. N. (2013). Meta-analysis of the effects of MI training on clinicians' behavior. J. Subst. Abuse Treat. 45, 155–162. 10.1016/j.jsat.2013.02.006 23537923

[B7] DuBois C. L. Astakhova M. N. DuBois D. A. (2013). Motivating behavior change to support organizational environmental sustainability goals, in Green Organizations: Driving change with IO psychology, eds Huffman A. H. Klein S. R. (New York, NY: Routledge), 186–207.

[B8] Forsberg L. Forsberg L. G. Lindqvist H. Helgason A. R. (2010). Research Clinician acquisition and retention of Motivational Interviewing skills: a two-and-a-half-year exploratory study. Subst. Abuse Treat. Prevent. Policy, 5, 1–14 10.1186/1747-597X-5-8 PMC2890671 20465805

[B9] Forsberg L. Wickström H. Källmén H. (2014). Motivational interviewing may facilitate professional interactions with inspectees during environmental inspections and enforcement conversations. PeerJ 2:e508. 10.7717/peerj.508 25177533 PMC4145070

[B10] Garabuau-Moussaoui I. (2014). How people work and live in energy-efficient workplaces? logics of actions, social tensions and actual issues of occupants in energy-efficient office buildings, in European Conference on Behavior and Energy Efficiency (Oxford, UK).

[B11] Gaume J. Bertholet N. Faouzi M. Gmel G. Daeppen J. B. (2010). Counselor motivational interviewing skills and young adult change talk articulation during brief motivational interventions. J. Subst. Abuse Treat. 39, 272–281. 10.1016/j.jsat.2010.06.010 20708900

[B12] Griesel C. (2004). Nachhaltigkeit im Bürokontext - eine partizipative Intervention zur optimierten Stromnutzung [Sustainability in organizational context - a participatory intervention to optimize energy user behavior]. Umweltpsychologie 8, 30–48.

[B13] Hannöver W. Blaut C. Kniehase C. Martin T. Hannich H. J. (2013). Interobserver agreement of the German translation of the motivational interviewing sequential code for observing process exchanges (MI-SCOPE; D). Psychol. Addict. Behav. 27, 1196–1200. 10.1037/a0033041 23772761

[B14] Hettema J. Steele J. Miller W. R. (2005). Motivational interviewing. Annu. Rev. Clin. Psychol. 1, 91–111 10.1146/annurev.clinpsy.1.102803.143833 17716083

[B15] Homburg A. (2004). Environmental actions in enterprises: a survey of influential factors and formation approaches from a social and environmental psychological perspective. Umweltpsychologie 8, 56–78.

[B16] Janda K. B. (2011). Buildings don't use energy: people do. Archit. Sci. Rev. 54, 15–22 10.3763/asre.2009.0050

[B17] Janis I. L. Mann L. (1977). Decision Making: A Psychological Analysis of Conflict, Choice, and Commitment. New York, NY: Free Press.

[B18] Kaplowitz M. D. Thorp L. Coleman K. Kwame Yeboah F. (2012). Energy conservation attitudes, knowledge, and behaviors in science laboratories. Energy Policy 50, 581–591 10.1016/j.enpol.2012.07.060

[B19] Kauran K. (2013). Motivation of Energy-Users is Crucial! Re-Co Newsletter 2013, 2, 5. Available online at: http://www.re-co.eu/sites/default/files/files/Re_Co_Newsletter_No2.pdf

[B20] Klonek F. E. Kauffeld S. (2012). Der Motivational Interviewing Skill Code, deutsche Version (2.3). [The motivational interviewing skill code, German version (2.3)]. Unpublished manual, Technische Universität Braunschweig. Available online at: https://www.tu-braunschweig.de/Medien-DB/aos/hinterlegte-pdfs/misc-d.pdf

[B21] Klonek F. E. Kauffeld S. (in press). !Providing Engineers with OARS and EARS: Effects of a Skills-Based Vocational Training in Motivational Interviewing for Engineers in Higher Education. Higher Education, Skills and Work-Based Learning.

[B22] Klonek F. E. Lehmann-Willenbrock N. Kauffeld S. (2014). Dynamics of resistance to change: a sequential analysis of change agents in action. J. Change Manag. 14, 334–360 10.1080/14697017.2014.896392

[B25] Kraft D. Neubeck S. (2004). Approaches of internal environmental communication within the scope of environmental management at Volkswagen Nutzfahrzeuge (VWN) Hannover. Umweltpsychologie 8, 42–55 10.1037/a0036227

[B26] Leyge C. (2014). Saving energy in the workplace: why, and for whom?, Presented at the European Conference on Behaviour and Energy Efficiency, (Oxford, UK).

[B27] Lokhorst A. M. Werner C. Staats H. van Dijk E. Gale J. L. (2013). Commitment and behavior change: a meta-analysis and critical review of commitment-making strategies in environmental research. Environ. Behav. 45, 3–34 10.1177/0013916511411477

[B28] Lombardi D. R. Button M. L. Westra H. A. (2014). Measuring motivation: change talk and counter-change talk in cognitive behavioral therapy for generalized anxiety. Cogn. Behav. Therapy 43, 12–21. 10.1080/16506073.2013.846400 24134594 PMC3863762

[B29] Lundahl B. W. Kunz C. Brownell C. Tollefson D. S. Burke B. (2010). A meta-analysis of motivational interviewing: twenty-five years of empirical studies. Res. Social Work Prac. 20, 137–160 10.1177/1049731509347850

[B30] Lutzenhiser L. (1993). Social and behavioral aspects of energy use. Annu. Rev. Energy Environ. 18, 247–289 10.1146/annurev.eg.18.110193.001335

[B31] Madson M. B. Loignon A. C. Lane C. (2009). Training in motivational interviewing: A systematic review. J. Subst. Abuse Treat. 36, 101–109. 10.1016/j.jsat.2008.05.005 18657936

[B32] Magill M. Apodaca T. R. Barnett N. P. Monti P. M. (2010). The route to change: within-session predictors of change plan completion in a motivational interview. J. Subst. Abuse Treat. 38, 299–305. 10.1016/j.jsat.2009.12.001 20149571 PMC2835844

[B33] Magill M. Gaume J. Apodaca T. R. Walthers J. Mastroleo N. R. Borsari B.. (2014). The technical hypothesis of motivational interviewing: a meta-analysis of MI's key causal model. J. Consult. Clin. Psychol. 82, 973–983. 10.1037/a0036833 24841862 PMC4237697

[B34] Martin T. Moyers T. B. Houck J. M. Christopher P. J. Miller W. R. (2005). Motivational Interviewing Sequential Code for Observing Process Exchanges (MI-SCOPE) Coder's Manual. Available online at: http://casaa.unm.edu/download/scope.pdf

[B35] Matthies E. (2000). Participatory planning - Considering the further development of a counseling environmental psychology. Umweltpsychologie 4, 84–99.

[B36] Miller W. R. Johnson W. R. (2008). A natural language screening measure for motivation to change. Addict. Behav. 33, 1177–1182. 10.1016/j.addbeh.2008.04.018 18558466

[B37] Miller W. R. Moyers T. B. Ernst D. Amrhein P. (2008). Manual for the Motivational Interviewing Skill Code (MISC): Version 2.1. Available online at: http://casaa.unm.edu/download/misc.pdf

[B38] Miller W. R. Rollnick S. (2013). Motivational Interviewing: Helping people change 3rd Edn New York, NY: Guilford Press.

[B39] Morgenstern P. (2014). Measuring changes in energy behaviours in complex non-domestic buildings, in Presented at the European Conference on Behaviour and Energy Efficiency, (Oxford, UK).

[B40] Motivational Interviewing Network of Trainers. (2008). Motivational Interviewing: Training for New Trainers. Resources for Trainers. Available online at: http://www.motivationalinterview.org/Documents/TNT_Manual_Nov_08.pdf

[B41] Moyers T. B. Martin T. B. Christopher P. J. Houck J. M. Tonigan J. S. Amrhein P. C. (2007). Client language as a mediator of motivational interviewing efficacy: where is the evidence? Alcohol. Clin. Exp. Res. 31(s3), 40–47s. 10.1111/j.1530-0277.2007.00492.x 17880345

[B42] Murtagh N. Nati M. Headley W. R. Gatersleben B. Gluhak A. Imran M. A. (2013). Individual energy use and feedback in an office setting: a field trial. Energy Policy 62, 717–728 10.1016/j.enpol.2013.07.090

[B43] Parkes G. (2014). Joint industry project on energy efficiency behaviour change, Presented at the European Conference on Behaviour and Energy Efficiency, (Oxford, UK).

[B44] Paulsen H. F. K. Klonek F. E. Rutsch F. Kauffeld S. (2015). Ready, steady, go! Veränderungsbereitschaft in der Interaktion messen [Ready, steady, go! Measuring readiness to change in interactions]. PERSONALquarterly, 2, 22–27.

[B45] Piderit S. K. (2000). Rethinking resistance and recognizing ambivalence: a multidimensional view of attitudes toward an organizational change. Acad. Manag. Rev. 25, 783–794 10.2307/259206

[B47] Rubak S. Sandbæk A. Lauritzen T. Christensen B. (2005). Motivational interviewing: a systematic review and meta-analysis. Br. J. Gen. Pract. 55, 305–312. 15826439 PMC1463134

[B48] Schäfer S. (2014). Intervention strategies and behavioural change at the workplace, Presented at the European Conference on Behaviour and Energy Efficiency, (Oxford, UK).

[B49] Steiner S. Diehl B. Engeser S. Kehr H. M. (2011). Sustainable innovations through user integration: implications for optimization of addressing users and designing innovation workshops. Umweltpsychologie 15, 52–70.

[B50] Tharan A. (2014). Climate protection by improved energy user behaviour at Eurpoean schools and public buildings, Presented at the European Conference on Behaviour and Energy Efficiency, (Oxford, UK).

