# Supplementary material for: Talking with consumers about energy reductions: recommendations from a motivational interviewing perspective
Source: Front Psychol. 2015 Mar 13;6:252. doi: 10.3389/fpsyg.2015.00252 (PMC4358062; doi:10.3389/fpsyg.2015.00252)
Supplement: Supplementary file 7 [file DataSheet1.DOCX]

### In this conversation, statements (volleys^[[1]](#footnote-1)^) were parsed into thought units (utterances). A volley can sometimes contain several thought units that require different MISC codes.

The MISC codes are written in the column Behavior Codes.

Target Behavior (Objective): Saving Energy

| Event | Speaker | Statement | Behavior Codes |
| --- | --- | --- | --- |
| 1 | Energy Manager: | [Today, I would like to talk with you about possibilites to save energy.] | **[Structure]** |
| 2 | Employee: | [Okay.] | **[Follow Neutral]** |
| 3 | Energy Manager: | [You work in a laboratory.  There are some options that will certainly allow you to save energy.] | **[Giving Information]** |
| 4 | Employee: | [I not only work in a laboratory, but I also work in an office.] [There are certainly some options to save energy.] [However, these so-called "options" are always connected to great expenditures.] | **[Follow Neutral]**  **[Change Talk-Other]**  **[Sustain Talk-Reason]** |
| 5 | Energy Manager: | [Don’t be so rash.] [First off, we should speak about the methods you already use to save energy.][Can you think of some?"] | **[Confrontation]**  **[Structure]**  **[Closed Question]** |
| 6 | Employee: | [Well, for example, I have set up my PC with a coupler strip so that it is not running on standby the entire time.] [But if I am in a hurry in the evenings, I don’t always remember to do this.] | **[Change Talk-Taking Steps]**  **[Sustain Talk-Taking Steps]** |
| 7 | Energy Manager: | [So it’s not so important to you to save energy in this way. I mean, it is a hand movement, then the switch is turned off.] | **[Confrontation]** |
| 8 | Employee: | [Well, it is often important,] [but when there is not time to do so, then there just isn’t time! And when I’m the only one doing this, it often doesn’t get done. All of my colleagues must do it in order for it to be effective.] | **[Change Talk-Other]**  **[Sustain Talk-Reason]** |
| 9 | Energy Manager: | [Well, because your colleagues don’t do it, you don’t do it either.] [But even when you are the only one doing it, it has an effect. You should really do this every night.] | **[Simple Reflection]**  **[Direct]** |
| 10 | Employee: | [Sure, then I end up crawling under my desk at closing time while all the others get to leave...] | **[Sustain Talk-Other]** |
| 11 | Energy Manager: | [You could keep an eye on the clock and five minutes before it’s time to quit, you would have time to turn off the switch, shut the windows, turn off the heating, turn off the lights, etc.] | **[Advise withouht permission]** |
| 12 | Employee: | [That sounds more like it would take a half hour, and I cannot do it. I do not have time for that.] | **[Sustain Talk-Reason]** |
| 13 | Energy Manager: | [And how do you do it in the mornings at home, when you are leaving your house or apartment?] | **[Open Question]** |
| 14 | Employee: | [Of course, I make sure to close all the windows, I flip the lights off, and so on...] | **[Change Talk-Taking Steps]** |
| 15 | Energy Manager: | [And why?] | **[Open Question]** |
| 16 | Employee: | [Obviously, it costs a lot of money,] [and when I am not home the entire day, I do not need the lights on.] | **[Change talk-Reason]**  **[Change talk-Reason]** |
| 17 | Energy Manager: | [You know that it costs money. Have you no conscience about how much money your employer must spend because you do not close your window in the evenings, you do not turn off the heating or turn off the switch?] | **[Closed Question]** |
| 18 | Employee: | [He has enough money, he shouldn’t be concerned!] | **[Sustain Talk-Reason]** |
| 19 | Energy Manager: | [But nevertheless, it would save energy and do good for the environment.] | **[Confrontation]** |
| 20 | Employee: | [Yes, this is true. I already know this.] [But there are other priorities with work.] [Naturally I can close the windows, switch off the PCs, etc.] | **[Change talk-Other]**  **[Sustain Talk-Reason]**  **[Change talk-Other]** |
| 21 | Energy Manager: | [I thought you do not have time?] | **[Closed Question]** |
| 22 | Employee: | [This has already been said.] [I can do these things,] [but in my eyes there just isn’t a large effect.] | **[Follow neutral]**  **[Change talk-Other]**  **[Sustain Talk-Reason]** |
| 23 | Energy Manager: | [And where do your priorities lie in your work?] | **[Open Question]** |
| 24 | Employee: | [The work must be done, the results have to be right, and it must go relatively quickly.] [I cannot just say, “Oh, the sun is going down, now I must drop everything and go turn off the lights and switch off everything.” Work processes are much too complex, and I would have to interrupt them to do this.] | **[Follow neutral]**  **[Sustain Talk-Need]** |
| 25 | Energy Manager: | [But you could, for example, ask another person to turn off the lights, someone who is perhaps closer to the switch and whose work flow would not be broken.] | **[Advise without permission]** |
| 26 | Employee: | [Yes, sure, I could do this.] [But two minutes later, when the sun has gone down, I must ask them again.] | **[Change talk-Other]**  **[Sustain Talk-Need]** |
| 27 | Energy Manager: | [Okay, perhaps this is too hard, because it is so variable as to when the sun goes down.] [But what, for example, about the lights in the office when you leave for the laboratory?] | **[Support]**  **[Open Question]** |
| 28 | Employee: | [I switch them off. I try to remember to do this. Sometimes I forget to do this, but this is only the exeception. | **[Change Talk-Taking steps]** |
| 29 | Energy Manager: | [This is still good.] | **[Affirm]** |
| 30 | Employee: | [Much of this is not feasible organizationally. For example, we have a separate freezer in each laboratory, where often there are only three test tubes inside.] [It would be more reasonable perhaps to have one large freezer to use where all the test tubes are stored.] [But that would just be a huge hassle because you would have to constantly walk from laboratory to laboratory.] [It would be worth it in terms of energy sparing,] [but in terms of our work, it would be a step backwards, because it is much more complicated and it would take more time.] | **[Sustain Talk-Need]**  **[Change Talk-Other]**  **[Sustain Talk-Reason]**  **[Change Talk-Reason]**  **[Sustain Talk-Reason]** |
| 31 | Energy Manager: | [So you think that there are capacities to save energy, but these are not implemented, because in terms of other aspects, there are too many drawbacks.] | **[Simple Reflection]** |
| 32 | Employee: | [Exactly. Who wants to go to the room thirty times a day to retrieve a test tube.] | **[Sustain Talk-Desire]** |
| 33 | Energy Manager: | [This is true.] [But maybe you could place the freezer in the hallway so that it is easily accessible to everyone.] | **[Support]**  **[Advise without permission]** |
| 34 | Employee: | [Then everyone would have to walk to it. The way it is now is just simpler. We did not buy all of these freezers for nothing; there is a purpose for it.] | **[Sustain Talk-Reason]** |
| 35 | Energy Manager: | [So you are not willing to do a few more steps to save energy, even though you know it would do this.] | **[Confrontation]** |
| 36 | Employee: | [Yes, exactly. I have said already that priorities are just elsewhere.] | **[Sustain Talk-Reason]** |

1. A volley is an uninterrupted sequence of utterances (thought units) by one party, before another party speaks [↑](#footnote-ref-1)
