# Supplementary material for: Talking with consumers about energy reductions: recommendations from a motivational interviewing perspective
Source: Front Psychol. 2015 Mar 13;6:252. doi: 10.3389/fpsyg.2015.00252 (PMC4358062; doi:10.3389/fpsyg.2015.00252)
Supplement: Supplementary file 8 [file DataSheet2.DOCX]

### In this conversation, statements (volleys^[[1]](#footnote-1)^) were parsed into thought units (utterances). A volley can sometimes contain several thought units that require different MISC codes.

The MISC codes are written in the column Behavior Codes.

Target Behavior (Objective): Saving Energy

| Event | Speaker | Statement | Behavior Codes |
| --- | --- | --- | --- |
| 1 | Energy Manager: | [Today, I would like to talk with you about possibilities to save energy.] | **[Structure]** |
| 2 | Employee: | [Okay.] | **[Follow neutral]** |
| 3 | Energy Manager: | [You work in a laboratory.  There are some options that will certainly allow you to save energy.] | **[Giving Information]** |
| 4 | Employee: | [I not only work in a laboratory, but I also work in an office.] [There are certainly some options to save energy.] [However, these so-called "options" are always connected to large expenditures.] | **[Follow Neutral]**  **[Change Talk-Other]**  **[Sustain Talk-Reason]** |
| 5 | Energy Manager: | [First we should perhaps talk about where you already save energy.] [What do you do already to save enery?] | **[Structure]**  **[Open Question]** |
| 6 | Employee: | [Well, I always turn on my PC using a coupler strip so that it is not always running on standby.] [But in the evenings if I am rushed before quitting time, I don’t always do this.] | **[Change Talk-Taking Steps]**  **[Sustain Talk-Taking Steps]** |
| 7 | Energy Manager: | [So often in the past you have switched the PC completely off, so that it does not run on standby. Occasionally, though, you are in too much of a hurry and this is not consistently done.] | **[Simple Reflection]** |
| 8 | Employee: | [Yes, I often pay attention to this.] [I find, however, that all of my colleagues must also do this for it to be effective.] | **[Change Talk-Taking Steps]**  **[Sustain Talk-Reason]** |
| 9 | Energy Manager: | [You believe that your colleagues should also think about turning their PC off completely in order to save more energy.] | **[Simple Reflection]** |
| 10 | Employee: | [Exactly! That would bring about a large effect.] | **[Change Talk-Other]** |
| 11 | Energy Manager: | [What would help to bring about having the PC turned off regularly in the future?] | **[Open Question]** |
| 12 | Employee: | [An idea would be for me to close up a bit early.] [Actually, I believe that I don’t have time for this,] [but I do it now anyway.] [The cost to my department to save energy is equal to my five minutes of work time – I guess.] | **[Change Talk-Other]**  **[Sustain Talk-Reason]**  **[Change Talk-Commitment]**  **[Change Talk-Reason]** |
| 13 | Energy Manager: | [You take a bit of time each evening before the office closes to remember to turn off the PC.] [What can you do further in order to save energy?] | **[Simple Reflection]**  **[Open Question]** |
| 14 | Employee: | [I don’t know... What is most important? Sure, I could make sure the windows are closed and that the lights are all off.] | **[Change Talk-Other]** |
| 15 | Energy Manager: | [So, in the future, you would like to turn off the PC, close the windows, and turn off the lights.] | **[Simple Reflection]** |
| 16 | Employee: | [Well, when I’m already at it, then it makes sense in this case.] [But whether or not I will always remember to do it is another question.] | **[Change Talk-Other]**  **[Sustain Talk-Ability]** |
| 17 | Energy Manager: | [You do not rightly know whether you will always remember.] | **[Simple Reflection]** |
| 18 | Employee: | [This is true. The issue is not yet a priority for me.] | **[Sustain Talk-Reason]** |
| 19 | Energy Manager: | [What can you do to help you to remember?] | **[Open Question]** |
| 20 | Employee: | [Mostly it is enough when I simply scribble a small note and hang it on my door. Then I can see it before I leave my office.] | **[Change Talk-Other]** |
| 21 | Energy Manager: | [If that works for you, then it is definitely the best solution.] | **[Emphasize Control]** |
| 22 | Employee: | [Yes, I am going to implement this right away.] | **[Change Talk-Commitment]** |
| 23 | Energy Manager: | [Great.] [You also mentioned that you work in a laboratory.] [What are you already doing there to save energy?] | **[Affirm]**  **[Simple Reflection]**  **[Open Question]** |
| 24 | Employee: | [Actually nothing, I don’t find it so important. I am not the woman in charge there.] | **[Sustain Talk-Reason]** |
| 25 | Energy Manager: | [So on the one hand, you save energy in the office, but on the other hand, the efficient use of energy in the laboratory is not important to you.] | **[Complex Reflection]** |
| 26 | Employee: | [Well, I wish to save energy when I can. If it didn’t matter to me, I would not do this in my office. There you are right.] [But I don’t want to interfere with the workflow of my colleagues.] | **[Change Talk-Desire]**  **[Sustain Talk-Desire]** |
| 27 | Energy Manager: | [You find it important to save energy in the laboratory as well. Your concern is that you would be disturbing your colleagues with it.] | **[Complex Reflection]** |
| 28 | Employee: | [Yes, in principle this is the task of the laboratory Manager. As soon as the laboratory Manager calls attention to it, then the others must also adhere to it.] [But this is true. Why do I put a note on my door and concern myself with my PC while in the laboratory each night, energy flows through. This shouldn’t be this way!] | **[Follow Neutral]**  **[Change Talk-Other]** |
| 29 | Energy Manager: | [You think that the laboratory Manager must actually say something and then your efforts to save energy would not be in vain.] | **[Simple Reflection]** |
| 30 | Employee: | [Yes, exactly. In principle, I only have to speak with Dr. Mueller about it. We have a team meeting anyway on Tuesday. There I can mention this. It is only a matter of turning off the lights when nobody is in the room, or closing the windows when the air conditioning is on.] [Some things, however, I simply cannot change.] [For example, in every room we have an extra freezer in order to store test tubes. In some instances, there are only two or three test tubes inside,] [and a somewhat larger freezer would do to store all the glassware.] [But naturally nobody wants to go into and out of a single room thirty times a day to retrieve test tubes.] | **[Change Talk-Need]**  **[Sustain Talk-Ability]**  **[Follow Neutral]**  **[Change Talk-Reason]**  **[Sustain Talk-Reason]** |
| 31 | Energy Manager: | [So you already believe that some things are possible and realizable to save energy in the laboratory, and others would hinder the work.] | **[Complex Reflection]** |
| 32 | Employee: | [Yes, it would not go over well if everyone had to make a huge effort because they all had to walk to another room. That also takes up time.] | **[Sustain Talk-Reason]** |
| 33 | Energy Manager: | [How important is it to you then that all of these freezers are replaced by just one?] | **[Open Question]** |
| 34 | Employee: | [Actually, I find it very important, otherwise I would not have brought it up.] [But I do not see a way to implement this.] | **[Change Talk-Desire]**  **[Sustain Talk-Ability]** |
| 35 | Energy Manager: | [At the moment you don’t have an idea about how your desire can be implemented.] | **[Simple Reflection]** |
| 36 | Employee: | [Exactly.] | **[Follow Neutral]** |
| 37 | Energy Manager: | [Can I perhaps make a suggestion?] | **[Advice with Permission]** |
| 38 | Employee: | [Yes, sure.] | **[Follow Neutral]** |
| 39 | Energy Manager: | [One possibility is that you simply open the topic for discussion on Tuesday. Perhaps your colleagues also have a solution to the problem or see things similarly.] | **[Giving Information]** |
| 40 | Employee: | [That is at least a possibility. I think with this suggestion I can perhaps get the ball rolling. I will in any case put this on our agenda!] | **[Change Talk-Commitment]** |

1. A volley is an uninterrupted sequence of utterances (thought units) by one party, before another party speaks [↑](#footnote-ref-1)
