# Supplementary material for: Talking with consumers about energy reductions: recommendations from a motivational interviewing perspective
Source: Front Psychol. 2015 Mar 13;6:252. doi: 10.3389/fpsyg.2015.00252 (PMC4358062; doi:10.3389/fpsyg.2015.00252)
Supplement: Supplementary file 9 [file DataSheet3.DOCX]

### In this conversation, statements (volleys^[[1]](#footnote-1)^) were parsed into thought units (utterances). A volley can sometimes contain several thought units that require different MISC codes.

The MISC codes are written in the column Behavior Codes.

Target Behavior (Objective): Saving Energy

| Event | Speaker | Statement | Behavior Codes |
| --- | --- | --- | --- |
| 1 | Energy Manager: | [Today, I would like to talk with you about possibilites to save energy.] | **[Structure]** |
| 2 | Employee: | [Okay.] | **[Follow Neutral]** |
| 3 | Energy Manager: | [You work in a laboratory. There are some options that will certainly allow you to save energy.] | **[Giving Information]** |
| 4 | Employee: | [I not only work in a laboratory, but I also work in an office.] [These so-called "options" are always connected to large expenditures.] | **[Follow Neutral]**  **[Sustain Talk-Reason]** |
| 5 | Energy Manager: | [Don’t be so rash.] [First off, we should speak about the methods you already use to save energy.] [Can you think of some?] | **[Confrontation]**  **[Structure]**  **[Closed Question]** |
| 6 | Employee: | [Well, for example, I have set up my PC with a coupler strip so that it is not running on standby the entire time.] [But if I am in a hurry in the evenings, I don’t always remember to do this.] | **[Change Talk-Taking Steps]**  **[Sustain Talk-Taking Steps]** |
| 7 | Energy Manager: | [So it’s not so important to you to save energy in this way. I mean, it is merely a hand movement, then the switch is turned off. That is really no big deal!] | **[Confrontation]** |
| 8 | Employee: | [In any case, there are more important things to consider. And if you find that it is no big deal, then you can come by in the evenings and turn off the PC.] | **[Sustain Talk-Other]** |
| 9 | Energy Manager: | [You should not push the responsibility on to others so easily. Everyone can do something about it so that we spend less money on energy.] | **[Confrontation]** |
| 10 | Employee: | [I see it just is not so simple, not while I have all my work to do.] | **[Sustain Talk-Other]** |
| 11 | Energy Manager: | [And how do you do it in the mornings at home, when you are leaving your house or apartment?] | **[Open Question]** |
| 12 | Employee: | [I make sure to close all the windows, I flip the lights off, and so on.] | **[Change Talk-Taking Steps]** |
| 13 | Energy Manager: | [And why only at home?”] | **[Open Question]** |
| 14 | Employee: | [Obviously, it costs a lot of money,] [and when I am not home the entire day, I do not need the lights on.] | **[Change Talk-Reason]**  **[Change Talk-Reason]** |
| 15 | Energy Manager: | [When it is your own money at stake, you will save energy, but when it is your employer’s money, it’s all the same to you.] [You should simply take the time before you leave to turn off your PC, close the window, and switch off the heating.] | **[Confrontation]**  **[Direct]** |
| 16 | Employee: | [And who pays me for this - Nobody!] | **[Sustain Talk-Reason]** |
| 17 | Energy Manager: | [You would be doing what is good for the environment and for the budget of the company.] | **[Confrontation]** |
| 18 | Employee: | [And what’s in it for me? Nothing!] | **[Sustain Talk-Reason]** |
| 19 | Energy Manager: | [Good feelings, a sense of environmentally conscious behavior.] | **[Confrontation]** |
| 20 | Employee: | [That doesn’t motivate me at all.] [But alright: I shut the lights off, I shut my PC off, I turn the heating off.] [Nevertheless, there are things that I simply cannot do!] | **[Sustain Talk-Desire]**  **[Change Talk-Other]**  **[Sustain Talk-Ability]** |
| 21 | Energy Manager: | [Then name some of those things for me please.] | **[Open Question]** |
| 22 | Employee: | [In my office I can always save energy, we have already spoken about this.] [But when we speak of the laboratory, there is nothing more I can do.] | **[Change Talk-Other]**  **[Sustain Talk-Other]** |
| 23 | Energy Manager: | [I cannot believe this. Especially in the laboratory is where the energy saving potential is even higher!] | **[Confrontation]** |
| 24 | Employee: | [Yes, but the priorities simply lie elsewhere.] | **[Sustain Talk-Reason]** |
| 25 | Energy Manager: | [Where do you believe the priorities lie in the laboratory?] | **[Closed Question}** |
| 26 | Employee: | [The work must be done, the results have to be right, and it must go relatively quickly.] [I cannot just say, “Oh, the sun is going down, now I must drop everything and go turn off the lights and switch off everything.” Work processes are much too complex, and I would have to interrupt them to do this.] | **[Follow Neutral]**  **[Sustain Talk-Reason]** |
| 27 | Energy Manager: | [But you could, for example, ask another person to turn off the lights, someone who is closer to the switch and whose work flow would not be broken.] | **[Advise without permission]** |
| 28 | Employee: | [And when the sun goes down, I must run those lights again. You know, a lot of this is simply not organizationally feasible.] [For example, we have a separate freezer in each laboratory, where often there is only three test tubes inside.] [It would be more reasonable perhaps to have one large freezer to use where all the test tubes are stored.] [But that would just be a huge hassle because you would have to constantly walk from laboratory to laboratory.] [It would be work in terms of energy sparing,] [but in terms of our work, it would be much too complicated and take up too much time.] | **[Sustain Talk-Reason]**  **[Follow Neutral]**  **[Change Talk-Other]**  **[Sustain Talk-Reason]**  **[Change Talk-Reason]**  **[Sustain Talk-Reason]** |
| 29 | Energy Manager: | [So you think that there are capacities to save energy, but these are not implemented, because in terms of other aspects, there are too many drawbacks.] | **[Simple Reflection]** |
| 30 | Employee: | [Who wants to go to the room thirty times a day to retrieve a test tube?] | **[Sustain Talk-Reason]** |
| 31 | Energy Manager: | [But perhaps someone can put the freezer in a central location, so that it is accessible for all your colleagues.] | **[Advise without permission]** |
| 32 | Employee: | [Then everyone would have to walk to it. The way it is now is just simpler. We did not buy all of these freezers for nothing; there is a purpose for it.] | **[Sustain Talk-Reason]** |
| 33 | Energy Manager: | [You would only have to go a few more steps. A little physical activity in the workplace never hurt no one. This way, you kill two birds with one stone.] | **[Advise without permission]** |
| 34 | Employee: | [I already get enough of a workout.] [As I’ve said, the priorities are simply elsewhere. We cannot change this and we will not change this.] | **[Follow Neutral]**  **[Sustain Talk-Commitment]** |

1. A volley is an uninterrupted sequence of utterances (thought units) by one party, before another party speaks [↑](#footnote-ref-1)
