# Supplementary material for: Talking with consumers about energy reductions: recommendations from a motivational interviewing perspective
Source: Front Psychol. 2015 Mar 13;6:252. doi: 10.3389/fpsyg.2015.00252 (PMC4358062; doi:10.3389/fpsyg.2015.00252)
Supplement: Supplementary file 10 [file DataSheet4.DOCX]

### In dieser Interaktion sind die folgenden Aussagen zum Teil in verschiedene Sinnabschnitte (d.h. Äußerungen) geteilt. Das heißt, dass manchmal eine *Aussage* auch mehrere *Äußerungen* beinhaltet.

| Event | Sprecher | Äußerungen | Verhaltenscode |
| --- | --- | --- | --- |
| 1 | Energiemanager: | [Heute möchte ich mit Ihnen darüber sprechen, welche Möglichkeiten Sie beim Energiesparen haben.] | **[Struktur]** |
| 2 | Mitarbeiter: | [Okay.] | **[Neutrales Folgen]** |
| 3 | Energiemanager: | [Sie arbeiten ja in einem Labor. Da gibt es sicher einige Möglichkeiten, wo man Energie einsparen kann.] | **[Informationen vermitteln]** |
| 4 | Mitarbeiter: | [Ich arbeite gar nicht nur in einem Labor, sondern auch in einem Büro.]  [Und sicherlich gibt es Möglichkeiten,]  [aber die sind dann direkt mit einem riesigen Aufwand verbunden.] | **[Neutrales Folgen]**  **[Change Talk-Aktivierung]**  **[Sustain Talk-Gründe]** |
| 5 | Energiemanager: | [Nicht so voreilig.][Erst einmal sollten wir darüber sprechen, was Sie schon fürs Energiesparen tun.]  [Fällt Ihnen da etwas ein?] | **[Konfrontation]**  **[Strukturierung]**  **[Geschlossene Frage]** |
| 6 | Mitarbeiter: | [Naja, ich schalte beispielsweise meinen PC immer mit so einer Steckleiste ganz aus, der läuft also nicht die ganze Zeit auf Standby.]  [Aber wenn ich abends vor dem Feierabend in Eile bin, mache ich es ab und zu auch mal nicht.] | **[Change Talk-Schritte]**  **[Sustain Talk-Schritte]** |
| 7 | Energiemanager: | [Also so wichtig ist es für Sie nicht, da Energie zu sparen. Ich meine, das ist ein Handgriff, den Schalter zu betätigen.] | **[Konfrontation]** |
| 8 | Mitarbeiter: | [Oft mache ich es ja auch,]  [aber wenn keine Zeit ist, dann ist keine Zeit! Und wenn nur ich das mache, bringt es doch sowieso auch nichts. Das muss dann schon jeder meiner Kollegen machen, damit das effektiv ist.] | **[Change Talk-Schritte]**  **[Sustain Talk-Gründe]** |
| 9 | Energiemanager: | [Also weil Ihre Kollegen es nicht machen, machen Sie das auch nicht.]  [Aber auch wenn nur Sie das machen, hat es einen Effekt. Sie sollten das wirklich jeden Abend machen.] | **[Einfache Reflexion]**  **[Anordnen]** |
| 10 | Mitarbeiter: | [Na klar, dann krabbel ich eben nach meinem Feierabend noch unter meinem Schreibtisch rum, während die anderen schon auf dem Weg nach draußen sind…] | **[Sustain Talk-Aktivierung]** |
| 11 | Energiemanager: | [Sie könnten ja auch einfach die Uhr im Auge behalten und fünf Minuten früher mit Ihrer Arbeit aufhören, sodass Sie noch Zeit haben, den Schalter zu betätigen, die Fenster zu schließen, die Heizung auszudrehen, das Licht auszumachen, usw.] | **[Ratschlag ohne Erlaubnis]** |
| 12 | Mitarbeiter: | [Das hört sich aber eher nach einer halben Stunde an, das mache ich nicht. Da habe ich gar keine Zeit für.] | **[Sustain Talk-Gründe]** |
| 13 | Energiemanager: | [Und wie machen Sie das morgens Zuhause, wenn Sie Ihr Haus oder Ihre Wohnung verlassen?] | **[Offene Frage]** |
| 14 | Mitarbeiter: | [Naja, da schließe ich schon vor dem Weggehen alle Fenster, mache das Licht aus und so…] | **[Change Talk-Schritte]** |
| 15 | Energiemanager: | [Und warum? | **[Offene Frage]** |
| 16 | Mitarbeiter: | [Naja, das kostet ja auch alles Geld]  [und wenn ich den ganzen Tag nicht Zuhause bin, braucht das Licht da nicht an sein.] | **[Change Talk-Gründe]**  **[Change Talk-Gründe]** |
| 17 | Energiemanager: | [Sie wissen also, dass es Geld kostet. Haben Sie gar kein schlechtes Gewissen, dass Ihr Arbeitgeber Geld bezahlen muss, weil Sie abends Ihre Fenster nicht schließen, Ihre Heizung nicht ausstellen oder den Schalter nicht drücken?] | **[Geschlossene Frage]** |
| 18 | Mitarbeiter: | [Der hat Geld genug, der soll sich nicht so anstellen!] | **[Sustain Talk-Gründe]** |
| 19 | Energiemanager: | [Aber nichtsdestotrotz würde es ja auch Energie sparen und so der Umwelt gut tun.] | **[Konfrontation]** |
| 20 | Mitarbeiter: | [Ja, das stimmt schon. Das weiß ich ja auch.]  [Aber bei der Arbeit liegen die Prioritäten eben einfach woanders.]  [Natürlich kann ich die Fenster schließen, den PC ausschalten usw.] | **[Change Talk-Aktivierung]**  **[Sustain Talk-Gründe]**  **[Change Talk-Aktivierung]** |
| 21 | Energiemanager: | [Ich dachte, dafür haben Sie keine Zeit?] | **[Geschlossene Frage]** |
| 22 | Mitarbeiter: | [Das war doch nur so dahin gesagt.]  [Ich kann es schon machen,]  [es hat in meinen Augen aber einfach keinen großen Effekt.] | **[Neutrales Folgen]**  **[Change Talk-Aktivierung]**  **[Sustain Talk-Gründe]** |
| 23 | Energiemanager: | [Und wo liegen Ihrer Meinung nach die Prioritäten bei der Arbeit?] | **[Offene Frage]** |
| 24 | Mitarbeiter: | [Die Arbeit muss fertig werden, die Ergebnisse müssen stimmen und es muss relativ zügig gehen.]  [Da kann ich mir nicht sagen ‚Oh, jetzt kommt die Sonne raus, jetzt muss ich aber rasch das Licht ausmachen‘ und alles stehen und liegen lassen und zum Schalter laufen. Dafür sind die Vorgänge viel zu komplex, die ich dafür unterbrechen müsste.] | **[Neutrales Folgen]**  **[Sustain Talk-Notwendigkeit]** |
| 25 | Energiemanager: | [Aber Sie könnten ja beispielsweise jemand anderes bitten, das Licht auszumachen, der sich vielleicht näher am Schalter befindet und seine Arbeit gerade sowieso unterbrochen hat.] | **[Ratschlag ohne Erlaubnis]** |
| 26 | Mitarbeiter: | [Ja stimmt, das könnte ich.]  [Aber zwei Minuten später, wenn die Sonne wieder weg ist, muss ich denjenigen dann wieder laufen lassen.] | **[Change Talk-Aktivierung]**  **[Sustain Talk-[Notwendigkeit]** |
| 27 | Energiemanager: | [Okay, vielleicht ist das auch schwierig, weil es sich so oft ändert, ob die Sonne da ist oder nun weg.]  [Aber was ist beispielsweise mit dem Licht in Ihrem Büro, wenn Sie ins Labor gehen?] | **[Unterstützung]**  **[Offene Frage]** |
| 28 | Mitarbeiter: | [Das schalte ich aus. Ich versuche zumindest, daran zu denken. Ab und zu vergesse ich es mal, aber das ist nur die Ausnahme.] | **[Change Talk-Schritte]** |
| 29 | Energiemanager: | [Das ist schonmal gut.] | **[Bestätigen]** |
| 30 | Mitarbeiter: | [Vieles ist organisatorisch aber auch einfach nicht machbar. Beispielsweise haben wir in jedem Labor einen eigenen Gefrierschrank, wo teilweise aber nur drei Reagenzgläser drin liegen.]  [Da wäre es schon sinnvoll, vielleicht einen größeren Gefrierschrank zu nutzen, wo dann alle Reagenzgläser drin gelagert werden.]  [Aber das wäre einfach ein riesiger Aufwand, weil man ständig von Labor zu Labor laufen müsste.]  [Das würde sich im Hinblick auf das Energiesparen lohnen,]  [aber im Hinblick auf unsere Arbeit wäre das ein Rückschritt, weil es viel aufwändiger ist und sicher auch mehr Zeit in Anspruch nimmt.] | **[Sustain Talk-Notwendigkeit]**  **[Change Talk-Aktivierung]**  **[Sustain Talk-Gründe ]**  **[Change Talk-Gründe]**  **[Sustain Talk-Gründe]** |
| 31 | Energiemanager: | [Sie denken also, dass es schon Kapazitäten zum Energiesparen gibt, diese aber einfach nicht umgesetzt werden, weil es im Hinblick auf andere Aspekte zu große Nachteile hätte.] | **[Einfache Reflexion]** |
| 32 | Mitarbeiter: | [Genau. Wer hat schon Lust, jeden Tag dreißig Mal den Raum zu wechseln, um ein Reagenzglas wegzubringen.] | **[Sustain Talk-Wunsch]** |
| 33 | Energiemanager: | [Ja, das stimmt schon.]  [Aber vielleicht könnte man den Gefrierschrank im Flur aufstellen, sodass er für jeden leicht erreichbar ist.] | **[Unterstützen]**  **[Ratschlag ohne Erlaubnis]** |
| 34 | Mitarbeiter: | [Trotzdem müssen dann alle laufen. Das ist so wie es jetzt ist eben einfacher. Man hat ja auch nicht umsonst so viele Gefrierschränke gekauft, das hat ja schon einen Sinn.] | **[Sustain Talk-Gründe]** |
| 35 | Energiemanager: | [Sie sind also nicht bereit, ein paar Schritte mehr zu tätigen um Energie zu sparen, obwohl Sie wissen, dass es das tun würde.] | **[Konfrontation]** |
| 36 | Mitarbeiter: | [Ja, genau. Wie gesagt, die Prioritäten liegen eben einfach woanders.] | **[Sustain Talk-Gründe]** |
