# Supplementary material for: Talking with consumers about energy reductions: recommendations from a motivational interviewing perspective
Source: Front Psychol. 2015 Mar 13;6:252. doi: 10.3389/fpsyg.2015.00252 (PMC4358062; doi:10.3389/fpsyg.2015.00252)
Supplement: Supplementary file 11 [file DataSheet5.DOCX]

### In dieser Interaktion sind die folgenden Aussagen zum Teil in verschiedene Sinnabschnitte (d.h. Äußerungen) geteilt. Das heißt, dass manchmal eine *Aussage* auch mehrere *Äußerungen* beinhaltet.

| Event | Sprecher | Äußerungen | Verhaltenscode |
| --- | --- | --- | --- |
| 1 | Energiemanager: | [Heute möchte ich mit Ihnen darüber sprechen, welche Möglichkeiten Sie beim Energiesparen haben.] | **[Strukturierung]** |
| 2 | Mitarbeiter: | [Okay.] | **[Neutrales Folgen]** |
| 3 | Energiemanager: | [Sie arbeiten ja in einem Labor. Da gibt es sicher einige Möglichkeiten, wo man Energie einsparen kann.] | **[Informationen vermitteln]** |
| 4 | Mitarbeiter: | [Ich arbeite ja nicht nur in einem Labor, sondern auch in einem Büro. ]  [Und sicherlich gibt es Möglichkeiten, ]  [aber die sind dann direkt mit einem gewissen Aufwand verbunden. ] | **[Neutrales Folgen]**  **[Change Talk-Aktivierung]**  **[Sustain Talk-Gründe]** |
| 5 | Energiemanager: | [Erst einmal sollten wir vielleicht darüber sprechen, wo sie schon Energie sparen.] [Was tun Sie denn bereits, um Energie zu sparen? ] | **[Strukturierung]**  **[Offene Frage]** |
| 6 | Mitarbeiter: | [Naja, ich schalte beispielsweise meinen PC immer mit so einer Steckleiste ganz aus, der läuft also nicht die ganze Zeit auf Standby. ]  [Aber wenn ich abends vor dem Feierabend in Eile bin, mache ich es ab und zu auch mal nicht. ] | **[Change Talk-Schritte]**  **[Sustain Talk-Schritte]** |
| 7 | Energiemanager: | [Sie haben also in der Vergangenheit schon sehr häufig darauf geachtet, den PC komplett auszuschalten, sodass er nicht auf Standby läuft. Ab und zu kommt es jedoch vor, dass Sie zu sehr in Eile sind und es dann ausnahmsweise nicht tun.] | **[Einfache Reflexion]** |
| 8 | Mitarbeiter: | [Ja, ich habe wirklich schon oft darauf geachtet.]  [Ich finde, aber, das muss schon jeder meiner Kollegen machen, damit das effektiv ist.] | **[Change Talk-Schritte]**  **[Sustain Talk-Gründe]** |
| 9 | Energiemanager: | [Sie sind der Meinung, dass Ihre Kollegen auch häufiger daran denken sollten, Ihren PC komplett auszuschalten, damit Sie noch mehr Energie sparen.] | **[Einfache Reflexion]** |
| 10 | Mitarbeiter: | [Genau! Das würde einen noch größeren Effekt bringen.] | **[Change Talk-Aktivierung]** |
| 11 | Energiemanager: | [Was würde Ihnen noch dabei helfen, den PC in Zukunft regelmäßig auszuschalten? ] | **[Offene Frage]** |
| 12 | Mitarbeiter: | [Eine Idee wäre, dass ich einfach etwas früher Schluss mache.]  [Eigentlich glaube ich, dass ich dafür keine Zeit habe,]  [aber die nehme ich mir ab jetzt einfach.] Die Kosten, die meine Abteilung mit dem Strom einspart, gleichen meine 5 Min. Personalkosten ja wahrscheinlich sogar aus.] | **[Change Talk-Aktivierung]**  **[Sustain Talk-Gründe]**  **[Change Talk-Selbstverpflichtung]**  **[Change Talk-Gründe]** |
| 13 | Energiemanager: | [Sie nehmen sich also jeden Abend bevor Sie das Büro verlassen einen festen Zeitraum, damit Sie daran denken, den PC auszuschalten.] [Was könnten Sie in dem Zuge noch tun, um Energie zu sparen?] | **[Einfache Reflexion]**  **[Offene Frage]** |
| 14 | Mitarbeiter: | [Weiß nicht... was könnte noch wichtig sein (*überlegt*…) Naja, ich könnte in dem Zuge gleich noch darauf achten, ob die Fenster geschlossen sind und ob das Licht ausgeschaltet ist.] | **[Change Talk-Aktivierung]** |
| 15 | Energiemanager: | [Also möchten Sie in Zukunft den PC ausschalten, die Fenster schließen und das Licht ausschalten.] | **[Einfache Reflexion]** |
| 16 | Mitarbeiter: | [Naja, wenn ich schon dabei bin, macht das doch auf jeden Fall Sinn.]  [Aber ob ich immer daran denke, ist noch mal eine andere Frage…] | **[Change Talk-Aktivierung]**  **[Sustain Talk-Fähigkeit]** |
| 17 | Energiemanager: | [Sie wissen nicht so recht, ob Sie auch immer daran denken werden.] | **[Einfache Reflexion]** |
| 18 | Mitarbeiter: | [Ist ja klar. Das Thema hat jetzt nicht oberste Priorität für mich.] | **[Sustain Talk-Gründe]** |
| 19 | Energiemanager: | [Was könnte Ihnen denn dabei helfen, daran zu denken?] | **[Offene Frage]** |
| 20 | Mitarbeiter: | [Meistens reicht es schon, wenn ich mir einfach ein kleines Zettelchen bastele und an meine Tür hänge. Das sehe ich dann, bevor ich mein Büro verlasse.] | **[Change Talk-Aktivierung]** |
| 21 | Energiemanager: | [Wenn das für Sie funktioniert, ist das sicherlich die beste Lösung.] | **[Autonomie des Klienten betonen]** |
| 22 | Mitarbeiter: | [Ja, das werde ich gleich umsetzen.] | **[Change Talk-Selbstverpflichtung]** |
| 23 | Energiemanager: | [Klasse.]  [Sie hatten auch erwähnt, dass sie in einem Labor arbeiten.] [Was tun Sie bereits dafür, um auch dort Energie zu sparen?] | **[Bestätigen]**  **[Einfach Reflexion]**  **[Offene Frage]** |
| 24 | Mitarbeiter: | [Eigentlich gar nichts, da ist es aber auch nicht so wichtig finde ich. Da bin ich ja nicht die Hauptverantwortliche.] | **[Sustain Talk-Gründe]** |
| 25 | Energiemanager: | [Also einerseits versuchen Sie, Energie in Ihrem Büro einzusparen, aber andererseits ist Ihnen der sparsame Umgang mit Energie im Laborgebäude gar nicht wichtig.] | **[Komplexe Reflexion]** |
| 26 | Mitarbeiter: | [Naja, ich möchte schon Energiesparen, wenn es geht. Ganz egal ist es mir nicht, sonst würde ich es ja auch in meinem Büro nicht machen. Da haben Sie schon Recht.  [Aber ich will den anderen nicht dazwischenfunken.] | **[Change Talk-Wunsch]**  **[Sustain Talk-Wunsch]** |
| 27 | Energiemanager: | [Sie finden es wichtig, auch im Labor Energie zu sparen, haben allerdings Bedenken, dass Sie Ihre Kollegen damit stören könnten.] | **[Komplexe Reflexion]** |
| 28 | Mitarbeiter: | [Ja, im Prinzip ist das die Aufgabe der Laborleitung. Sobald der Laborleiter darauf aufmerksam macht, müssen sich auch die anderen daran halten.]  [Aber es stimmt schon. Warum klebe ich mir einen Zettel an die Tür und achte auf meinen PC, während im Labor jede Nacht die Abzüge durchlaufen. Das geht eigentlich gar nicht!] | **[Neutrales Folgen]**  **[Change Talk-Aktivierung]** |
| 29 | Energiemanager: | [Sie denken, eigentlich müsste nur der Laborleiter mal etwas sagen und dann wären Ihre Bemühen Energie zu sparen nicht völlig umsonst.] | **[Einfache Reflexion]** |
| 30 | Mitarbeiter: | [Ja genau. Im Prinzip muss ich nur mal mit Herrn Dr. Müller sprechen. Wir haben am Dienstag sowieso eine Teambesprechung. Da kann ich ihn kurz vorher ja mal abfangen. Es geht ja nur darum, das Licht auszuschalten, wenn niemand im Raum ist oder die Fenster zu schließen, wenn die Klimaanlage läuft.]  [Manches werde ich allerdings einfach nicht ändern können.]  [Zum Beispiel haben wir in jedem Raum einen extra Gefrierschrank, um darin Reagenzgläser zu lagern. Teilweise sind da nur zwei bis drei Gläser drin,]  [da würde ein etwas größerer Gefrierschrank reichen, in dem alles gelagert wird.]  [Aber es hat natürlich niemand Lust, am Tag dreißig Mal den Raum zu wechseln, um ein Reagenzglas wegzubringen.] | **[Change Talk-Notwendigkeit]**  **[Sustain Talk-Fähigkeit]**  **[Neutrales Folgen]**  **[Change Talk-Gründe]**  **[Sustain Talk-Gründe]** |
| 31 | Energiemanager: | [Sie glauben also schon, dass einige Dinge machbar und umsetzbar sind, um im Labor Energie zu sparen, andere jedoch die Arbeit behindern würden.] | **[Komplexe Reflexion]** |
| 32 | Mitarbeiter: | [Ja, das geht ja nicht, dass ein Riesenaufwand entsteht, weil alle in andere Räume laufen müssen. Das nimmt ja auch Zeit in Anspruch.] | **[Sustain Talk-Gründe]** |
| 33 | Energiemanager: | [Wie wichtig ist es für Sie denn, dass die vielen Gefrierschränke durch einen einzigen ersetzt werden?] | **[Offene Frage]** |
| 34 | Mitarbeiter: | [Eigentlich finde ich es schon wichtig, sonst hätte ich es ja nicht angesprochen.]  [Aber ich sehe leider keinen Weg, das umzusetzen.] | **[Change Talk-Wunsch]**  **[Sustain Talk-Fähigkeit]** |
| 35 | Energiemanager: | [Momentan haben Sie noch keine Idee, wie Sie Ihren Wunsch umsetzen können.] | **[Einfache Reflexion]** |
| 36 | Mitarbeiter: | [Genau.] | **[Neutrales Folgen]** |
| 37 | Energiemanager: | [Kann ich vielleicht einen Vorschlag machen?] | **[Ratschalg mit Erlaubnis]** |
| 38 | Mitarbeiter: | [Ja, klar.] | **[Neutrales Folgen]** |
| 39 | Energiemanager: | [Eine Möglichkeit ist, dass Sie das einfach offen in der Besprechung am Dienstag ansprechen. Vielleicht haben Ihre Kollegen ja auch eine Lösung für das Problem oder sehen das genauso…] | **[Informationen vermitteln]** |
| 40 | Mitarbeiter: | [Das ist zumindest eine Möglichkeit. Ich denke, mit diesem Vorschlag kann ich vielleicht erstmal einen Stein ins Rollen bringen. Ich werde es auf jeden Fall mal mit in die Runde nehmen!] | **[Change Talk-Selbstverpflichtung]** |
