# Supplementary material for: Talking with consumers about energy reductions: recommendations from a motivational interviewing perspective
Source: Front Psychol. 2015 Mar 13;6:252. doi: 10.3389/fpsyg.2015.00252 (PMC4358062; doi:10.3389/fpsyg.2015.00252)
Supplement: Supplementary file 12 [file DataSheet6.DOCX]

### In dieser Interaktion sind die folgenden Aussagen zum Teil in verschiedene Sinnabschnitte (d.h. Äußerungen) geteilt. Das heißt, dass manchmal eine *Aussage* auch mehrere *Äußerungen* beinhaltet.

| Event | Sprecher | Äußerungen | Verhaltenscode |
| --- | --- | --- | --- |
| 1 | Energiemanager: | [Heute möchte ich mit Ihnen darüber sprechen, welche Möglichkeiten Sie zum Energiesparen haben.] | **[Strukturierung]** |
| 2 | Mitarbeiter: | [Okay.] | **[Neutrales Folgen]** |
| 3 | Energiemanager: | [Sie arbeiten ja in einem Labor. Da gibt es doch sicherlich einige Möglichkeiten, wo man Energie einsparen kann.] | **[Informationen vermitteln]** |
| 4 | Mitarbeiter: | [Ich arbeite gar nicht nur in einem Labor, sondern auch in einem Büro.]  [Und die sogenannten ‚Möglichkeiten‘ sind immer direkt mit einem riesigen Aufwand verbunden.] | **[Neutrales Folgen]**  **[Sustain Talk-Gründe]** |
| 5 | Energiemanager: | [Nicht so voreilig.][Erst einmal sollten wir darüber sprechen, was Sie schon fürs Energiesparen tun.] [Fällt Ihnen da etwas ein?] | **[Konfrontation]**  **[Strukturierung]**  **[Geschlossene Frage]** |
| 6 | Mitarbeiter: | [Naja, ich schalte beispielsweise meinen PC immer mit so einer Steckleiste aus, der läuft also nicht die ganze Zeit auf Standby.]  [Aber wenn ich abends vor dem Feierabend in Eile bin, mache ich es ab und zu auch mal nicht.] | **[Change Talk-Schritte]**  **[Sustain Talk-Schritte]** |
| 7 | Energiemanager: | [Also so wichtig ist es anscheinend für Sie nicht, da Energie zu sparen. Ich meine, das ist doch bloß ein Handgriff, den Schalter zu betätigen. Das ist ja nun wirklich kein großer Aufwand!] | **[Konfrontation]** |
| 8 | Mitarbeiter: | [Es gibt auf jeden Fall wichtigere Dinge. Und wenn Sie finden, dass es kein großer Aufwand ist, können Sie ja abends in mein Büro kommen und den PC ausschalten.] | **[Sustain Talk-Aktivierung]** |
| 9 | Energiemanager: | [Sie sollten die Verantwortung nicht so einfach von sich auf andere schieben. Jeder kann etwas dafür tun, damit wir weniger Geld für Energie ausgeben.] | **[Konfrontation]** |
| 10 | Mitarbeiter: | [Ich sehe es aber einfach nicht ein, das alles während meiner Arbeitszeit zu tun.] | **[Sustain Talk-Aktivierung]** |
| 11 | Energiemanager: | [Und wie machen Sie das zum Beispiel morgens Zuhause, wenn Sie ihr Haus oder Ihre Wohnung verlassen?] | **[Offene Frage]** |
| 12 | Mitarbeiter: | [Da schließe ich schon vor dem Weggehen alle Fenster, mache das Licht aus und so…] | **[Change Talk-Schritte]** |
| 13 | Energiemanager: | [Und warum nur zu Hause?] | **[Offene Frage]** |
| 14 | Mitarbeiter: | [Naja, das kostet ja auch alles Geld] [und wenn ich den ganzen Tag nicht Zuhause bin, braucht das Licht da nicht die ganze Zeit an sein.] | **[Change Talk-Gründe]**  **[Change Talk-Gründe]** |
| 15 | Energiemanager: | [Wenn es um Ihr eigenes Geld geht, sparen Sie also Energie, aber wenn es das Geld Ihres Arbeitgebers ist, ist es Ihnen völlig egal.] [Sie sollten sich einfach die Zeit nehmen, vor dem Feierabend Ihren PC auszuschalten, das Fenster zu schließen, die Heizung auszustellen usw.] | **[Konfrontation]**  **[Anordnen]** |
| 16 | Mitarbeiter: | [Und wer bezahlt mir das - Niemand!] | **[Sustain Talk-Gründe]** |
| 17 | Energiemanager: | [Sie tun was für die Umwelt und sparen darüberhinaus das Budget Ihrer Firma.] | **[Konfrontation]** |
| 18 | Mitarbeiter: | [Und was springt für mich dabei raus? Nichts!] | **[Sustain Talk-Gründe]** |
| 19 | Energiemanager: | [Ein gutes Gefühl springt dabei raus und ein umweltbewusstes Verhalten!] | **[Konfrontation]** |
| 20 | Mitarbeiter: | [Das treibt mich nun gar nicht an.]  [Aber nun gut: Ich schalte das Licht aus, ich schalte meinen PC aus, ich drehe die Heizung runter.]  [Trotzdem gibt es Dinge, die ich einfach nicht ändern kann!] | **[Sustain Talk-Wunsch]**  **[Change Talk-Aktivierung]**  **[Sustain Talk-Fähigkeit]** |
| 21 | Energiemanager: | [Dann nennen Sie mir die Dinge doch mal.] | **[Offene Frage]** |
| 22 | Mitarbeiter: | [In meinem Büro kann ich schon Energiesparen, das haben wir ja auch gerade besprochen.]  [Aber spätestens wenn es um das Labor geht, kann ich nichts mehr tun.] | **[Change Talk-Aktivierung]**  **[Sustain Talk-Aktivierung]** |
| 23 | Energiemanager: | [Das kann ich mir nicht vorstellen. Gerade im Labor ist das Einsparpotential noch viel höher!] | **[Konfrontation]** |
| 24 | Mitarbeiter: | [Ja. Aber da liegen die Prioritäten einfach woanders.] | **[Sustain Talk-Gründe]** |
| 25 | Energiemanager: | [Wo liegen denn Ihrer Meinung nach die Prioritäten im Labor?] | **[Geschlossene Frage]** |
| 26 | Mitarbeiter: | [Die Arbeit muss fertig werden, die Ergebnisse müssen stimmen und es muss relativ zügig gehen.]  [Da kann ich mir nicht sagen ‚Oh, jetzt kommt die Sonne raus, jetzt muss ich aber rasch das Licht ausmachen‘ und alles stehen und liegen lassen und zum Schalter laufen. Dafür sind die Vorgänge viel zu komplex, die ich dafür unterbrechen müsste.] | **[Neutrals Folgen]**  **[Sustain Talk-Gründe]** |
| 27 | Energiemanager: | [Aber Sie können ja jemand anderes bitten, das Licht auszumachen, der sich näher am Schalter befindet und seine Arbeit gerade sowieso unterbrochen hat.] | **[Ratschlag ohne Erlaubnis]** |
| 28 | Mitarbeiter: | [Und wenn die Sonne wieder weg is, muss ich denjenigen dann wieder laufen lassen. Wissen Sie, vieles ist organisatorisch einfach nicht machbar.]  [Beispielsweise haben wir in jedem Labor einen eigenen Gefrierschrank, wo teilweise aber nur drei Reagenzgläser drin liegen.]  [Da wäre es schon sinnvoll, vielleicht einen größeren Gefrierschrank zu nutzen, wo dann alle Reagenzgläser drin gelagert werden.]  [Aber das wäre einfach ein riesiger Aufwand, weil man ständig von Labor zu Labor laufen müsste.]  [Das würde sich im Hinblick auf das Energiesparen lohnen,]  [aber im Hinblick auf unsere Arbeit wäre es viel aufwändiger und nimmt zu viel Zeit in Anspruch.] | **[Sustain Talk-Gründe]**  **[Neutrales Folgen]**  **[Change Talk-Aktivierung]**  **[Sustain Talk-Gründe]**  **[Change Talk-Gründe]**  **[Sustain Talk-Gründe]** |
| 29 | Energiemanager: | [Sie denken also, dass es schon Kapazitäten zum Energiesparen gibt, diese aber einfach nicht umgesetzt werden, weil es im Hinblick auf andere Aspekte zu große Nachteile hätte.] | **[Einfache Reflexion]** |
| 30 | Mitarbeiter: | [Wer hat schon Lust, jeden Tag dreißig Mal den Raum zu wechseln, um ein Reagenzglas wegzubringen.] | **[Sustain Talk-Wunsch]** |
| 31 | Energiemanager: | [Aber vielleicht könnte man den Gefrierschrank zentral in den Flur stellen, sodass er für jeden Kollegen gut erreichbar ist.] | **[Ratschlag ohne Erlaubnis]** |
| 32 | Mitarbeiter: | [Trotzdem müssen dann alle laufen. Das ist so wie es jetzt ist eben einfacher. Man hat ja auch nicht umsonst so viele Gefrierschränke gekauft, das hat ja schon einen Sinn.] | **[Sustain Talk-Gründe]** |
| 33 | Energiemanager: | [Sie müssten doch nur ein paar Schritte mehr gehen. Ein wenig körperliche Betätigung würden Ihnen bestimmt auch gut tun. So schlagen sie gleich zwei Fliegen mit einer Klappe.] | **[Ratschlag ohne Erlaubnis]** |
| 34 | Mitarbeiter: | [Ich mach schon genug Sport…]  [Wie gesagt, die Prioritäten liegen eben einfach woanders.] [Wir können da nichts ändern und wir werden da auch nichts ändern.] | **[Neutrales Folgen]**  **[Sustain Talk-Selbstverfplichtung]** |
